# Effects of g-C_3_N_4_ on bacterial community and tetracycline resistance genes in two typical sediments in tetracycline pollution remediation

**DOI:** 10.3389/fmicb.2022.964401

**Published:** 2022-09-16

**Authors:** Xuemei Hu, Xiaoyong Chen, Yao Tang, Zhenggang Xu, Yelin Zeng, Yonghong Wang, Yunlin Zhao, Yaohui Wu, Guangjun Wang

**Affiliations:** ^1^College of Life Science and Technology, Central South University of Forestry and Technology, Changsha, China; ^2^College of Arts and Sciences, Governors State University, University Park, IL, United States; ^3^Key Laboratory of National Forestry and Grassland Administration on Management of Western Forest Bio-Disaster, College of Forestry, Northwest A&F University, Yangling, China

**Keywords:** tetracycline, g-C_3_N_4_, bacterial community, metagenomic analysis, TRGs, functional analysis

## Abstract

Photocatalysis, as a novel technique, has been widely used for antibiotic pollution remediation in wastewater. In the processes of degradation and removal of antibiotics, the impact of photocatalysts on microenvironment is very important but remains poorly understood. In the present study, the effect of typical photocatalyst g-C_3_N_4_ (Graphitic carbon nitride) on microbial community was investigated in two sediment types (riverbed sediment and pig-farm sediment) polluted by tetracycline (TC) in central southern China. The riverbed sediment and pig farm sediment samples were respectively exposed to g-C_3_N_4_ (25, 75, 125 mg⋅kg^–1^) and TC (60, 120, 180 mg⋅L^–1^) treatments alone or combination for 30 days, respectively. The bacterial community and antibiotic resistance genes (ARGs) of the treated sediments were analyzed by Illumina sequencing and metagenomic sequencing. Studies had shown that: TC, g-C_3_N_4_, and TC/g-C_3_N_4_ have significant effects on the changes of microbial communities and components in riverbed sediment, but they do not exist in pig farm sediment. The most alterations of microbial taxa were *Acidobacteriota*, *Actinobacteriota*, and *Desulfobacterota* in riverbed sediment, and *Elusimicrobiota* in the pig farm sediment under various treatments. Through network analysis, it was found that the distribution of microorganisms in the pig farm sediment is more complex and more stable. The addition of g-C_3_N_4_ reduced the absolute abundance of ARGs in the two examined sediments, but not significantly changed their relative abundance of ARGs. The g-C_3_N_4_ application was beneficial to the removal of TC residues and to the prevention of the generation and transmission of ARGs in sediments. Our results suggested that g-C_3_N_4_ was a suitable photocatalyst with excellent application prospect for the removal of TC residues and the control of ARGs in environment.

## Introduction

Antibiotics had been used worldwide for disease treatment in livestock and aquaculture as feed additives in the past decades (Henriksson et al., 2018). Over time, these substances would flow into the surrounding soil in various ways, thus causing serious pollution to the environment and causing great harm to the human body (Zhan et al., 2021). Tetracycline (TC) is one of the most inexpensive classes of antibiotics and it had been widely used as veterinary therapy in livestock and fish farming industry in the world (Gao et al., 2020). Due to its extensive usage and poor bioavailability, relative high concentrations of TC residues have been found in various environments (Mahmoud and Abdel-Mohsein, 2019). The presence of TC residues could inhibit microbial growth and reduce the number of bacteria in sediment (He et al., 2021), cause negative effects on the structure and activity of environmental microbiota in aquaculture (Martinez-Porchas and Vargas-Albores, 2017), and affect soil ecological functions in terrestrial ecosystems (Santas-Miguel et al., 2020). In some cases, TC could induce new antibiotic resistance mutations by promoting microbial communities to defend the attack of antibiotics, developing recombination and repair function (Zhang L. L. et al., 2021).

As a novel remediation way, photocatalysts have been applied for the remediation of a variety of organic pollutants (Guo et al., 2021). By inducing strongly oxidizing reagents they could decompose some pollution substances present in the atmosphere (Nath et al., 2016; Sun et al., 2022a). Owing to numerous active sites, photocatalysts could degrade organic pollutants in air and water (Shi et al., 2022). Many photocatalysts have been applied to remove emerging water pollutants such as drug pollution and shown a remarkable stability in the photocatalytic degradation (Tobajas et al., 2017). Recently, photocatalysts have been applied to remove some pathogenic bacteria in the environment, such as *Escherichia coli*, *Salmonella typhimurium* and *Staphylococcus aureus* (Tang et al., 2019; Zhang C. et al., 2019). Photocatalysis were considered as excellent ecofriendly candidates for water remediation given their outstanding photostability, and minimal ecotoxicological effects on aquatic biota (Tobajas et al., 2017; Serra et al., 2020). Photocatalysts might influence antibiotic resistant bacteria (ARB) and antibiotic resistance genes (ARGs) under photocatalytic conditions, so that it might be a worth-trying material for antibiotic resistance control (Guo and Tian, 2019). Among various photocatalysts, graphite carbon nitride (g-C_3_N_4_) was a kind of unalloyed polymer with good thermal, chemical and electrochemical stability (Hou et al., 2020). Thus, it has a great potential for applying in the field of catalytic elimination of environmental pollutants (Jun et al., 2015), such as degradation of TC in sewage and sludge samples (Yan et al., 2020; Zhao et al., 2020).

Sediment systems were essential components when assessing aquatic ecosystems functionality (He et al., 2019), in which microorganisms were the most abundant group of organisms in sediment for maintaining sediment biological activity and reducing pollutant levels (Yan et al., 2020). Several studies have been conducted to examine the impact of application of g-C_3_N_4_ on removal of TC in environments, but the effect of g-C_3_N_4_ usage on microenvironments in TC contaminated sediments was still poorly understand (Gao et al., 2021; Guo et al., 2021). It is particular true in riverbed sediment and pig farm sediment, were less was known about the influence of g-C_3_N_4_ on the richness and diversity of ARGs in the TC polluted environments.

This study aimed to examine the influence of g-C_3_N_4_ application on the ARGs and the structure of bacterial community in TC contaminated riverbed sediments and pig farm sediments. The specific objectives of this study were: (a): to evaluate the ability of g-C_3_N_4_ to remove TC pollutants in the examined sediments, (b): to analyze the effects of TC, g-C_3_N_4_ and TC/g-C_3_N_4_ treatments on bacterial community structure in the different sediments, (c): to assess the effects of g-C_3_N_4_ on ARGs and tetracycline resistance genes (TRGs) in the two sediment types, and (d): to reveal the interrelation and interaction among the TRGs, bacterial species communities and functional metabolism of bacteria in the studied environments. The results from this study would provide theoretical basis and scientific reference for better understanding of photocatalyst application in the degradation of antibiotics in environments.

## Materials and methods

### Sediment sampling and physicochemical parameters

**FIGURE 1:**
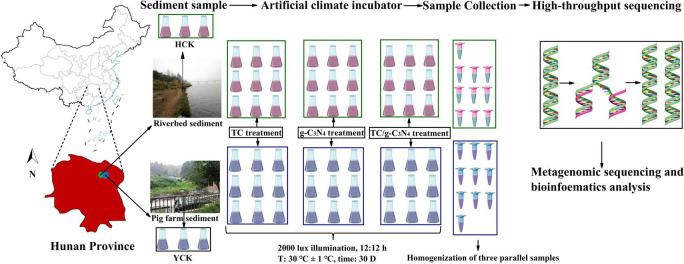
Location of the study site, with sediment sampling and experimental design.

The pig farm sediments in this experiment were collected from several farms located in Liuyang County, Hunan Province of China (113°51′55′′E, 28°28′08′′N) (Figure 1). These selected farms have complete manure treatment facilities, where the treated effluents were released into receiving water-collectors and the deposited manures were applied as fertilizers to the surrounding agricultural lands. About 185.3 ng⋅kg^–1^ was still detected in the pig farm sediment, which had a serious risk of TC contamination. The riverbed sediments were collected from the Xiangjiang River in Changsha, Hunan Province (112°94′71′′E, 28°14′23′′N), and about 17.46 ng⋅kg^–1^ TC residue was detected in the river bed sediment, which also had a certain pollution risk. The field sampling work was carried out in July 2020.

The sediment samples were manually taken from the surface sediment (10–20 cm in depth) at five points on a 2 m^2^ rectangular area, with one point at the center and other four points at the corners. The five samples were pooled to form a mixed sample for a rectangular area. There were five replicated mixed samples (2 kg each) for each of the two sediment types.

Two types of sediment were characterized to determine pH, Total nitrogen (TN), ammonium nitrogen (NH_4_^+^-N), nitrate nitrogen (NO_3_^–^-N), Total phosphorus (TP) and Total potassium (TK). The pH of the sediment was determined by a composite electrode (pH glass-saturated mercury) in a sediment/water ratio of 1:2.5. TN, NH_4_^+^-N, and NO_3_^–^-N contents were analyzed using an Elemental analyzer (Vario MACRO cube, Elementar, Germany). TP content was analyzed using the sodium hydroxide fusion-Molybdenum-antimony resistance colorimetric method. TK was determined by flame photometry (Flame photometer 410, sherwood). The physical properties of the sediment were shown in the Supplementary Table 1.

The TC residue in each sample was detected by high performance liquid chromatography (HPLC) according to the method. The standard substances of TC (C_22_H_24_N_2_O_8_⋅HCl, purity, USP grade, purity 98%) were purchased from Shanghai Aladdin Biochemical Technology (Shanghai, China). TC standard stock solution (1000 mg⋅L^–1^) was prepared with HPLC grade methanol (Shimadzu Corporation, Kyoto, Japan) and stored at −20°C in the dark. The mobile phase contained 0.1% trifluoroacetic acid and acetonitrile gradient. The flow rate was 1 mL⋅min^–1^, the equilibrium solution was 10% B solution, and the gradient rose to 30% B solution within 20 min at a flow rate of 1 mL⋅min^–1^ and a temperature of 35°C. Three samples with the same label were used to measure recoveries of TC in the samples respectively, and it was in the range of 95.53–105.27. Values of 3 and 10 were used as signal-to-noise ratios for measurement limit and quantization limit. The relative standard deviations of TC content in each sample was within 10%. The quantitative standard curve of the measured antibiotics and the confirmation of the measurement method are shown in Supplementary Figure 1 and the Supplementary Table 2.

### Experimental design, and sampling

#### Tetracycline and g-C_3_N_4_

1 g TC was dissolved in 100 mL sterile water to form a TC solution with a concentration of 10 mg⋅mL^–1^. Then, the solution was diluted to different TC concentrations (60, 120, 180 mg⋅L^–1^) with sterile water. Bulk g-C_3_N_4_ were obtained by direct polycondensation of melamine according to the procedure (Hou et al., 2020). Typically, a certain amount of melamine was loaded into a porcelain boat with a lid and placed in a tube furnace, then heated up to 550°C for 4 h, after cooled to room temperature naturally, the resultant powder was collected for further use (Liu E. L. et al., 2021; Chen et al., 2022).

Tetracycline was extracted according to the method of Zhang et al. (2015), within 24 h after the sample collection. 2 g of sediment sample was acidified to pH 4.0 by adding HCl, followed by addition of 40 mL of McIlvaine-Na_2_ EDTA buffer (0.2 M) and acetonitrile (v:v = 1:2) through 60 s vortex for three times, and ultra-sonicated at room temperature (15 min at 300 mA). After centrifugation in air-cooled conditions at 10,000 rpm for 10 min, the supernatant was collected from the mixture. The combined supernatant was then concentrated to about 5 mL and diluted to 100 mL with Milli-Q water. The samples were extracted using Oasis HLB (150 mg, 6 mL) extraction cartridges. The extracts percolated through the adsorbent at a flow rate of approximately 1.0 mL⋅min^–1^. Afterward, the column was rinsed with 5 mL of ultra-pure water and 5 mL of methanol (5%), dried under vacuum for 10 min, and eluted twice with 5 mL of methanol-ethyl acetate solution (10%, 1:9, v:v). The selected analytes were dried under a gentle nitrogen stream, re-dissolved in 1 mL of mobile phase [acetonitrile: formic-acid (0.1%), 1:9, v:v]. Final extracts were transferred to 2 mL amber vials for HPLC analysis.

#### Sediment exposure design

Four experimental treatments were conducted for each of the pig farm sediment and the riverbed sediment and they were: (1) Control treatments (CK), (2) TC treatments, (3) g-C_3_N_4_ treatments, and (4) TC/g-C_3_N_4_ treatments. In CK, 100 g of pig farm sediment or riverbed sediment were added to 100 mL of sterile water and labeled as YCK or HCK. In the TC treatments, 100 g sediment samples were added to 100 mL of TC solutions with different concentrations (60, 120, 180 mg⋅L^–1^). The treatments for pig farm sediment were named as YT_*L*_, YT_*M*_, and YT_*H*_, and the treatments for riverbed sediment as HT_*L*_, HT_*M*_, and HT_*H*_, respectively. In the g-C_3_N_4_ treatments, 100 g sediment samples were mixed with g-C_3_N_4_ to different concentration (25, 75, 125 mg⋅kg^–1^). The treatments for pig farm were labeled as YP_*L*_, YP_*M*_, and YP_*H*_, and the treatments for riverbed sediment as HP_*L*_, HP_*M*_, and HP_*H*_, respectively. In TC/g-C_3_N_4_ treatments, 100 g sediment samples were added to 100 mL of solution with various TC/g-C_3_N_4_ combination (60 mg⋅L^–1^/25 mg⋅kg^–1^, 120 mg⋅L^–1^/75 mg⋅kg^–1^, 180 mg⋅L^–1^/125 mg⋅kg^–1^). The treatments for pig farm were assigned as YT_*L*_P_*L*_, YT_*M*_P_*M*_, YT_*H*_P_*H*_, and the treatments for riverbed sediment as HT_*L*_P_*L*_, HT_*M*_P_*M*_, and HT_*H*_P_*H*_, respectively. There were a total of 20 treatments (two groups of control + two sediment samples × 9 treatment conditions) were pretreated and each treatment was performed for three replications (Figure 1). All samples were placed in a homeothermic incubator (RGX-350) at 30 ± 1°C under 2000 lux illumination with a light dark period of 12:12 h. After treated for 30 days, 1–2 g sediment sample was taken and stored in a refrigerator at -80°C for the analysis of bacterial community structure, diversity and ARGs.

### DNA extraction, library construction, and metagenomic sequencing

#### DNA extraction and 16S rRNA gene sequencing

Total genomic DNA of each sediment sample was extracted from 2 g of sediment samples using the FastDNA Spin Kit (Omega Bio-tek, Norcross, GA, United States) for pig farm sediment. The DNA extract was checked on 1% agarose gel, and DNA concentration and purity were determined with NanoDrop 2000 UV-vis spectrophotometer (Thermo Scientific, Wilmington, United States). The hypervariable region V3-V4 of the bacterial 16S rRNA gene was amplified with primer pairs 338F (5′-ACTCCTACGGGAGGCAGCAG-3′) and 806R (5′-GGACTACHVGGGTWTCTAAT-3′) by an ABI GeneAmp 9700 PCR thermocycler (ABI, CA, United States). The details of PCR were fully described in Supplementary materials.

#### Illumina MiSeq sequencing

Majorbio Bio-Pharm Technology Co., Ltd., (Shanghai, China) pooled purified amplicons in equimolar amounts and paired-end sequenced on an Illumina MiSeq PE300 platform (Illumina, San Diego, United States) using standard protocols.

#### Processing of sequencing data

Fastp (Version 0.20.0) demultiplexed and quality-filtered the raw 16S rRNA gene sequencing readings before merging them with FLASH (Version 1.2.7) (Magoč and Salzberg, 2011; Chen et al., 2018). Using unique barcodes, sequencing reads were assigned to each sample and shortened by removing the primer sequence and barcode. The original DNA fragments were merged into tags using FLASH (Magoč and Salzberg, 2011). According to QIIME (Version 1.9.1) quality-controlled process, the raw tags was filtered under specific filtering conditions to generate high-quality clean tags (Caporaso et al., 2010).

In order to generate effective tags, the UCHIME algorithm was used to remove the chimeric sequence from the clean tags on the basis of reference to each operational taxonomic unit (OTU). Assign each remaining sequence to an OTU when at least 97% of the threshold identity was obtained using UPARSE software (Version 7.0.1) and chimeric sequence were identified and deleted (Edgar, 2013). According to the 16S rRNA database (Silva v138), each OTU representative sequence was classified by RDP Classifier version 2.2 with a confidence interval of 0.7 (Wang et al., 2007).

#### Metagenomic sequencing and bioinformatics analysis

Metagenomic shotgun sequencing libraries of the four selected sediment samples were performed by Majorbio, Inc., (Shanghai, China) using Illumina MiSeq PE300 platform. This approach generated 245,754,330 raw reads and 35,398,741,165 total clean bases for the four samples (HCK, YCK, HT_*H*_P_*H*_, and YT_*H*_P_*H*_). MEGAHIT was used to clean and assemble the sequence, contigs with length of 300 bp or more selected as the final assembling result, and then contigs were used for further gene prediction and annotation.

### Statistical analysis

The statistical analysis of biological information of OTU was performed by using Uparse software. The Bray–Curtis distance was used to generate principal co-ordinates analysis (PCoA) for the visualization of complex multidimensional data. Drawing rarefaction curve and bacterial community composition were determined by R package vegan (Version 3.3.3). Annotation of antibiotic resistance was conducted using Diamond against Comprehensive Antibiotic Resistance Database (CARD, Version 3.0.9) (Buchfink et al., 2015). The correlation coefficient of Spearman rank among the bacteria phylum in the two sediment types was calculated using Network software. Significantly positive correlation (*p* < 0.05) was used to build network in Cytoscape (Version 3.5.2) in Prefuse Force Directed Layout by betweenness centrality (correlation coefficient > 0.5). The One-way ANOVA analyses were conducted using SPSS (IBM SPSS Statistics 26.0).

## Results

### Characterizations

**FIGURE 2:**
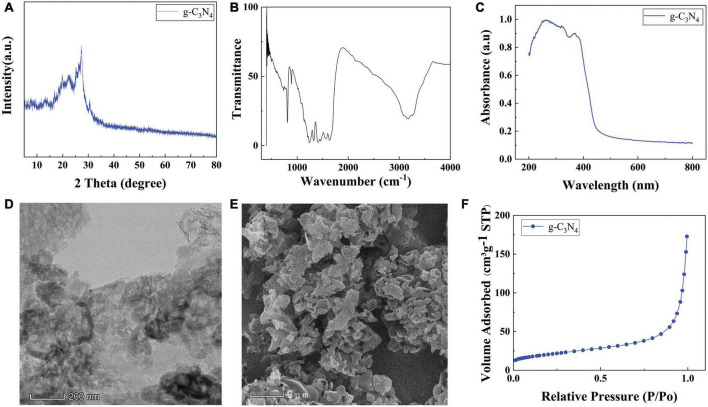
**(A)** XRD of g-C_3_N_4_, FT-IR **(B)** patterns of the g-C_3_N_4_, **(C)** UV-vis SEM images of **(D)** g-C_3_N_4_, TEM images of **(E)** g-C_3_N_4_, **(F)** Nitrogen adsorption-desorption isotherms of g-C_3_N_4_.

As shown in Figure 2A, a strong peak appeared in the diffraction pattern of g-C_3_N_4_ at 2θ = 27.8°, corresponding to the (002) planes of g-C_3_N_4_, which is the characteristic interlayer stacking peak of g-C_3_N_4_. Meanwhile, a weak peak appeared in the diffraction pattern of g-C_3_N_4_ at 2θ = 12.8°, corresponding to the (100) planes of g-C_3_N_4_. The FT-IR spectra of the g-C_3_N_4_ was shown in Figure 2B, sharp absorption peaks at about 800 cm^–1^ of g-C_3_N_4_ was assigned to the stretching vibration modes of the triazine units. The absorption bands at 1251, 1325, and 1437 cm^–1^ are attributed to the typical stretching vibration modes of the C = N heterocycles. Figure 2C shown the UV-vis diffuse reflectance spectra to characterize the optical properties of the g-C_3_N_4_ sample, g-C_3_N_4_ nanosheets showed strong absorption both in the UV and in the visible light regions. The morphology and structure of the g-C_3_N_4_ samples were investigated using SEM and TEM technology. Figure 2D shown TEM images of g-C_3_N_4_ photocatalysts. The g-C_3_N_4_ presented a fold Nano thin layer. From the SEM image of Figure 2E, it can be seen that g-C_3_N_4_ is made of multiple nanosheets stacked on top of each other, with a typical stacked layer structure. BET analysis of g-C_3_N_4_ photocatalyst The N_2_ adsorption-desorption isotherms and BET specific surface area (SSA) of the g-C_3_N_4_ photocatalyst were shown in Figure 2F. g-C_3_N_4_ has a mesoporous structure, which facilitates the reduction of mass transfer limitations and collecting light in the photocatalytic process.

### Removal of tetracycline in different sediments

**FIGURE 3:**
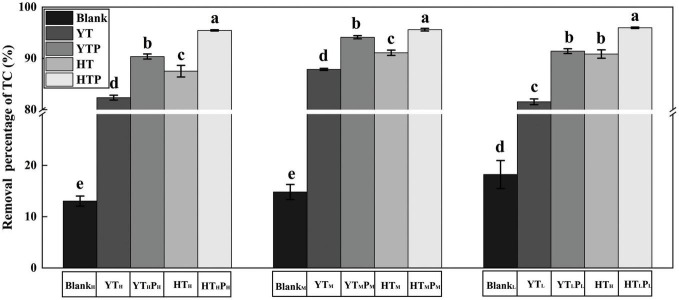
Removal percentage of TC residues from various treated sediments. Blank_*H*_, Blank_*M*_, and Blank_*L*_ refer to 180, 120, and 60 mg⋅L^– 1^ TC solution after stored for 30 days, respectively. HT, HTP refer to the riverbed sediment treated by TC and TC/g-C_3_N_4_ for 30 days, respectively. YT, YTP refer to the pig farm sediment treated by TC and TC/g-C_3_N_4_ for 30 days, respectively. The error bars represent the SDs (*n* = 3). Significant differences were determined by Duncan’s test There were significant differences between different letters (a, b, c, d, e) (*p* < 0.05).

After stored for 30 days, the removal percentage of TC residues was less than 20% in the blank group with different TC concentrations (Figure 3). The removal proportion of TC residues significantly increased in both of pig farm sediment (YT) and riverbed sediment (HT) when compared to the blank group (*p* < 0.05). The residue of TC in pig farm sediment was significantly higher (*p* < 0.05) than that in riverbed sediment after 30 days exposure. Adding g-C_3_N_4_ promoted TC removal in the two examined sediment types.

### Bacterial diversity in the riverbed sediment and pig farm sediment

A total of 604,406 high-quality readings (average 30,203) were obtained from 20 samples, after eliminating low and short quality reads, replicates, singletons, and chimeras (Supplementary Figure 2). The number of OTU varied for different groups and a total of 8,934 OTUs was detected out in all samples (Supplementary Table 3). There were 1,696 OTUs identified in YCK, and the observed OTUs ranged from 1701 to 2026 in the 9 pig farm sediment samples treated by different concentrations of TC, g-C_3_N_4_ and TC/g-C_3_N_4_. Moreover, 3,118 OTUs were obtained from HCK, and the range of OTUs was from 3304 to 3850 OTUs in the 9 riverbed sediment samples treated by different level of TC, g-C_3_N_4_ and TC/g-C_3_N_4_.

The Shannon indices were 5.07, 4.98, and 5.03 for the three kinds of treated pig farm sediments (YT, YP, and YTP), respectively. The mean value was 5.03 ± 0.045, similar to that of the CK sediment (YCK: 5.09). The Simpson index of bacteria in YT, YP, and YTP were not significantly different compared to that in YCK, implying that the addition of TC, g-C_3_N_4_ or TC/g-C_3_N_4_ had no significant effect on the diversity and richness of microbial community in pig farm sediment. The treated riverbed sediments, HT, HP, and HTP, had a Shannon index of 6.86, 6.85, and 6.85, respectively. The mean value was 6.85 ± 0.005, which was higher than that of the CK sediment (HCK: 6.59). The Simpson index of bacteria was higher in HT, HP, and HTP treatments than in HCK. The results implied that the addition of TC, g-C_3_N_4_, TC/g-C_3_N_4_ increased the diversity and richness of microbial communities of the riverbed sediments.

**FIGURE 4:**
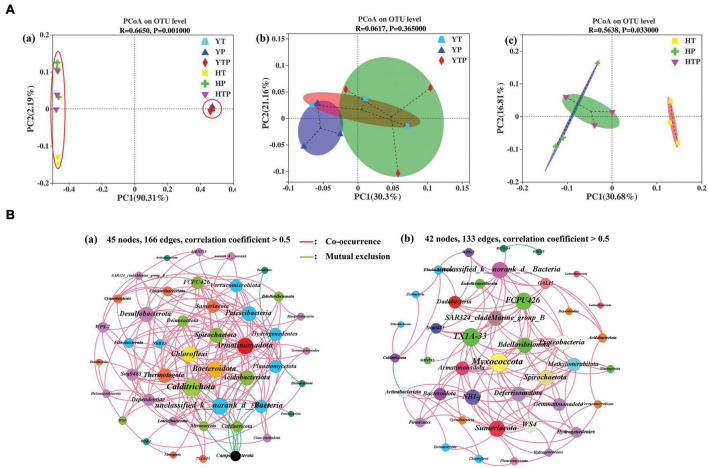
Principal Coordinate Analysis (PCoA) and co-occurrence network analysis illustrates the dissimilarities and stability of microbiome. **(A)** PCoA illustrate dissimilarities of bacterial communities’ structure, (a) refer to PCoA among two types of sediment system contain of TC, g-C_3_N_4_, TC/g-C_3_N_4_, respectively, (b) refer to PCoA of the bacterial communities in pig farm sediment system contain of TC, g-C_3_N_4_, TC/g-C_3_N_4_, respectively, (c) refer to PCoA of the bacterial communities in riverbed sediment system contain of TC, g-C_3_N_4_, TC/g-C_3_N_4_, respectively. YT, YP, YTP refer to the pig farm sediment treated with different concentrations of TC, g-C_3_N_4_ and TC/g-C_3_N_4_ for 30 days, respectively; HT, HP, HTP refer to the riverbed sediment treated with different concentrations of TC, g-C_3_N_4_ and TC/g-C_3_N_4_ for 30 days, respectively. **(B)** The co-occurrence network analysis of bacterial community at phylum level in pig farm sediment (a) and riverbed sediment (b) (correlation coefficient > 0.5). The nodes of each network are colored according to phylum affiliation (97%) and sized according to degree of connection. The edges connecting the nodes are represented by red lines to indicate co-occurrence interactions or green to indicate mutualistic exclusions.

Principal co-ordinates analysis was used to investigate the differences of bacterial community between the two sorts of sediment (Figure 4Aa), which explained 90.31 and 2.19% at the first two coordinates. The pig farm sediment treated by TC, g-C_3_N_4_ and TC/g-C_3_N_4_ clustered nearby axis-2, respectively (*p* < 0.05), while the riverbed sediment treated by TC, g-C_3_N_4_ and TC/g-C_3_N_4_ clustered along axis-1. Further PCoA analysis for the pig farm sediment samples revealed that after being treated by TC, g-C_3_N_4_ and TC/g-C_3_N_4_. The bacterial community in the pig farm sediment samples did not change significantly under the TC, g-C_3_N_4_ and TC/g-C_3_N_4_ treatments (*p* > 0.05) (Figure 4Ab), in which the first two axes (PCoA1 and PCoA2) explained 30.30 and 21.16% of the total variance, respectively. However, the addition of TC, g-C_3_N_4_ and TC/g-C_3_N_4_ lead an obvious separation of the bacterial community in the riverbed sediment samples (*p* < 0.05) (Figure 4Ac), in which the first two axes (PCoA1 and PCoA2) explained 30.68 and 16.81% of the total variance in the bacterial communities, respectively. These results suggested that the difference of the bacterial community composition between pig farm sediment and riverbed sediment samples depended on the sediment properties rather than the treatment method. Each sediment type had the capacity to form its specific ecosystem with an independent clustering. Additionally, the effect of TC, g-C_3_N_4_ and TC/g-C_3_N_4_ application on the microbial communities structure was more obvious in riverbed sediment than in pig farm sediment.

Analysis of co-occurrence network was performed based on the phylum level (Figure 4B). The numbers of nodes and edges were 45 and 166, and 42 and 133 in pig farm sediment and riverbed sediment, respectively. The network of the pig farm sediment was more complex and the stability of the bacterial community structure was higher in pig farm sediment than in riverbed sediment, which was beneficial to resist the changes in the bacterial community caused by external environment. Therefore, TC treatment samples in pig farm sediment groups was clearly separated from either those from g-C_3_N_4_ treatment or TC/g-C_3_N_4_ treatment (Figure 4Ab).

### Microbial community of the pig farm sediment and riverbed sediment

**FIGURE 5:**
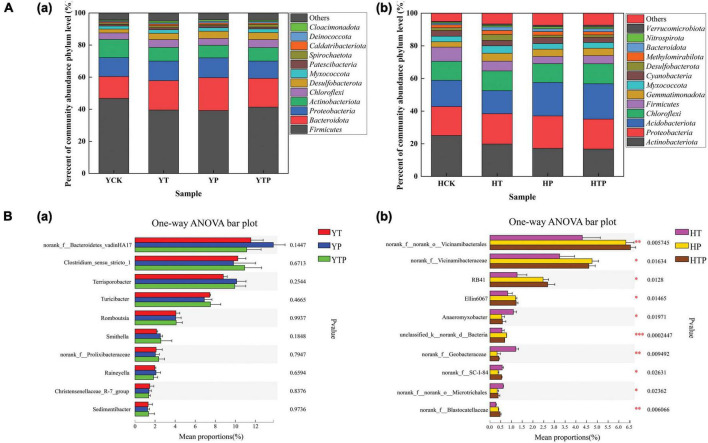
Dynamics of the bacterial community under different treatment. **(A)** Relative abundance of bacterial taxa at the phylum level in pig farm sediment and riverbed sediment. **(B)** The One-way ANOVA to compare bacterial community structure changes, (a) Bacterial community structure changes at genus level in pig farm sediment, (b) Bacterial community structure changes at genus level in riverbed sediment. YCK refer to the pig farm sediment for 30 days, HCK refer to the riverbed sediment for 30 days. *p < 0.05, **p < 0.01, ***p < 0.001.

The dominant bacterial phyla were *Firmicutes, Proteobacteria, Actinobacteriota, Bacteroidota* in the pig farm sediment samples and there was no significant difference in species abundance and genus level among different sample treatments (Figures 5Aa,Ba and Supplementary Table 5). However, bacterial communities were changed significantly within 4 treated groups in the riverbed sediments. The dominant bacteria were *Actinobacteriota*, *Proteobacteria*, *Acidobacteriota*, *Chloroflexi* in riverbed sediment (Figure 5Ab and Supplementary Table 6), which were different from those in pig farm sediment. As the abundance of *Acidobacteriota* increased in HP and HTP groups, the addition of g-C_3_N_4_ significantly stimulated the growth of *Acidobacteriota* species, such as *RB41* in the riverbed sediment (Figure 5Bb).

### Abundance and diversity of ARGs types and tetracycline resistance genes subtypes

There were 2,415,418,884 high-quality reads after filtering in the four representative metagenomics libraries (YCK, HCK, YT_*H*_P_*H*_, and HT_*H*_P_*H*_) (Supplementary Table 7). A total of 836 ARGs subtypes potentially conferring resistance to 20 classes of antibiotics were detected in the two sediment types. Multidrug resistance genes were most abundant (39.19%) with 199 subtypes observed, followed by TRGs, with 60 subtypes that comprised 12.74% of abundance, and MLS resistance genes with 90 subtypes that comprised 11.33% of abundance (Supplementary Figure 3).

**FIGURE 6:**
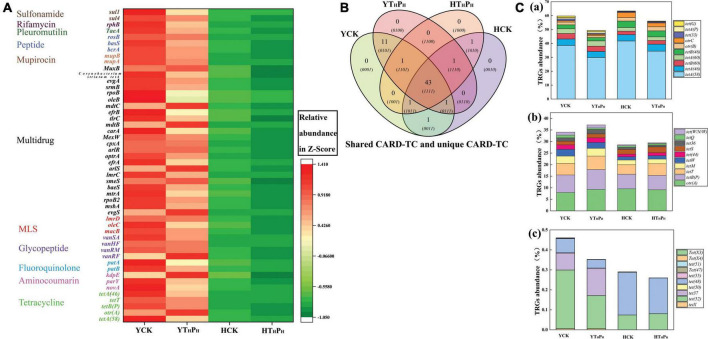
**(A)** Heatmap showing abundances of 50 background ARGs in 4 sediments. **(B)** Shared TRGs and unique TRGs. **(C)** Relative abundances of TRGs among sediment of ecosystems, (a) Efflux pump genes, (b) Ribosomal protection protein genes, (c) TC inactivation genes. YCK means the pig farm sediments alone treated for 30 days, HCK means the riverbed sediments alone treated for 30 days, YT_*H*_P_*H*_ means the pig farm sediment system contain 180 mg⋅L^– 1^ of TC and 125 mg⋅kg^– 1^ of g-C_3_N_4_ for 30 days, HT_*H*_P_*H*_ means the riverbed sediment system contain 180 mg⋅L^– 1^ of TC and 125 mg⋅kg^– 1^ of g-C_3_N_4_ for 30 days.

The pig farm sediment samples encoded ARGs belonging to 80.48% of antibiotic classes found in these environments while riverbed sediment samples only encoded 19.52% of antibiotic classes (Supplementary Table 8). The ARGs abundance were higher in pig farm sediment than in riverbed sediment, implying a more complex composition of antibiotic resistance mechanisms existed in the pig farm sediment (Figure 6A). The top 50 selected ARGs subtypes accounted for 99.00% of the 20 total ARGs types identified across the four samples, and used to analyze the effect of g-C_3_N_4_ on the abundance of ARGs in the samples (Figure 6A and Supplementary Table 8). Four genes [*tetA(58)*, *tetB(P)*, *tetT*, and *tetA(46)*] conferring resistance to TC also had higher abundant in the YCK and HCK than in the YT_*H*_P_*H*_ and HT_*H*_P_*H*_, respectively. There were 45 ARGs subtypes conferring resistance to other antibiotics in the top 50 selected ARGs subtypes, e.g., *macB*, *evgS*, *novA*. The ARGs subtypes were less in g-C_3_N_4_ treatment groups (YT_*H*_P_*H*_, HT_*H*_P_*H*_) than those in CK groups (YCK, HCK), especially in pig farm sediment (Figure 6A and Supplementary Table 9). The g-C_3_N_4_ could reduce the abundance of most ARGs in sediment environment. TC/g-C_3_N_4_ treatment not only decreased the abundance of TRGs, but also the occurrence and diversity of non-TC ARGs, such as Multidrug resistance genes and MLS resistance genes.

A total of 60 TRGs subtypes potentially conferring resistance to TC were detected out in the four sediment samples, most belonging to antibiotic efflux (34 TRGs subtypes), antibiotic inactivation (13 TRGs subtypes), and antibiotic target protection (13 TRGs subtypes). The shared and unique TRGs subtypes were examined by Venn diagrams for sediment samples (Figure 6B). 43 TRGs subtypes were shared by 4 sediments, accounting for 71.7% of TRGs abundance across all samples. Additionally, 11 TRGs subtypes were shared by 2 pig farm sediment samples (YCK, YT_*H*_P_*H*_), however, only 1 TRGs subtypes was shared by 2 riverbed sediments samples (HCK, HT_*H*_P_*H*_).

Much of the TRGs abundance was contributed by the gene components of efflux pump complexes, such as *tetA(58)*, *tetA(46)*, *tetB(60)*, and *tetA(60)* (Figure 6C). Interestingly, *tetA(58)* had higher abundance than *tetA(46)*, *tetB(60)*, and *tetA(60)* by an order of magnitude. Compared to YCK and HCK, the percentage of *tetA(58)* decreased respectively from 37.59 and 41.77% to 29.91 and 34.44% in YT_*H*_P_*H*_ and HT_*H*_P_*H*_, suggesting that g-C_3_N_4_ could decrease the abundance of *tetA(58)* in the environment, but there was no significant effect on other low abundance TRGs.

**FIGURE 7:**
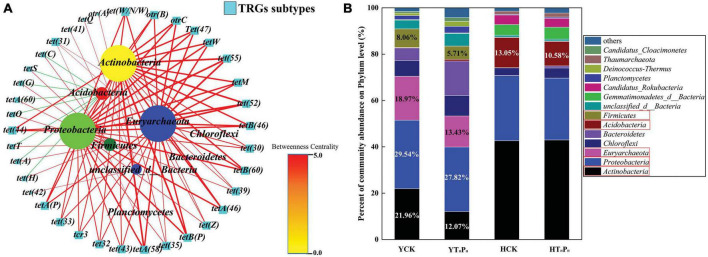
Network analyses among the TRGs, samples, and bacterial community across: YCK, HCK, YT_*H*_P_*H*_, and HT_*H*_P_*H*_. **(A)** TRGs-bacterial community network analyses. Red and green edges represent the positive and negative correlations, respectively. The network graphs were displayed with Cytoscape 3.5.2 in Prefuse Force Directed Layout by Betweenness centrality. **(B)** Distribution histograms of the bacteria with TRGs at the phylum levels. YCK means the pig farm sediments alone treated for 30 days, HCK means the riverbed sediments alone treated for 30 days, YT_*H*_P_*H*_ means the pig farm sediment system contain 180 mg⋅L^– 1^ of TC and 125 mg⋅kg^– 1^ of g-C_3_N_4_ for 30 days, HT_*H*_P_*H*_ means the riverbed sediment system contain 180 mg⋅L^– 1^ of TC and 125 mg⋅kg^– 1^ of g-C_3_N_4_ for 30 days.

Networking plots were constructed to figure out the TRGs that were significantly correlated (correlation coefficient > 0.5) with dominant bacteria (*p* < 0.05) (Figure 7A). The *tetA(58)*, *tetA(46)*, *tetB(60)*, and *tetB(46)* were the dominant genes in the samples and were positively related with *Proteobacteria*, *Actinobacteria*, *Euryarchaeota*, *Acidobacteria*, *Firmicutes*, *Planctomycetes*, *Chloroflexi*, *unclassified_d__Bacteria*. The genes of And *tet(C)*, *tet(H)*, *tet(T)*, *tet(S)*, *tet(H)*, *tetA(60)*, and *tet(A)* were negatively related with *Acidobacteria*. Thus, the elimination of TRGs by g-C_3_N_4_ in different sediments might attributed to the influence of g-C_3_N_4_ on their bacteria community structure. g-C_3_N_4_ reduced the relative abundance of bacteria with TRGs, such as *Actinobacteria* (21.96–12.07%), *Proteobacteria* (29.54–27.82%), *Euryarchaeota* (18.94–13.43%), and *Firmicutes* (8.06–5.71%) in the pig farm sediment. Similarly, g-C_3_N_4_ also reduced the relative abundance of bacteria with TRGs, such as *Acidobacteria* (13.05–10.58%) in the riverbed sediment (Figure 7B).

### Bacterial metabolic functional analysis

**FIGURE 8:**
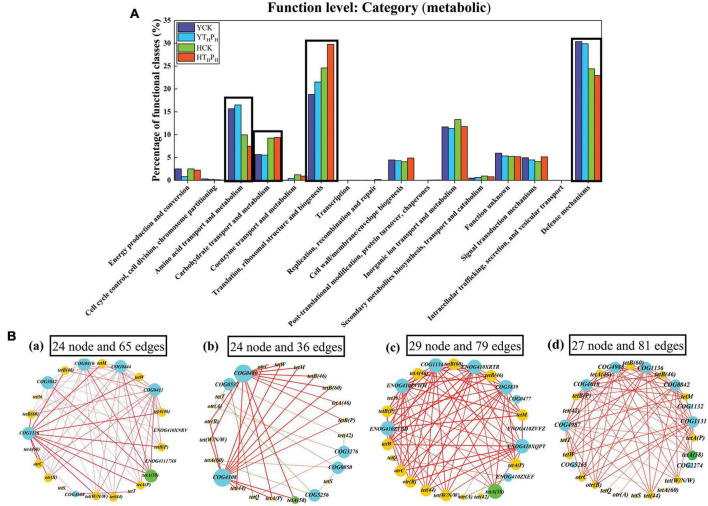
Bacterial metabolic functional analysis. **(A)** Metabolic functional traits and categories of the four sediment samples according to the eggNOG database. **(B)** Correlation network analysis of TRGs and the predominant metabolic functional, (a) Correlation network analysis of TRGs and “Amino acid transport and metabolism,” (b) Correlation network analysis of TRGs and “Carbohydrate transport and metabolism,” (c) Correlation network analysis of TRGs and “Translation, ribosomal structure and biogenesis,” (d) Correlation network analysis of TRGs and “Defense mechanism.” The network graphs were displayed with Cytoscape 3.5.2 in Prefuse Force Directed Layout by betweenness centrality (correlation coefficient > 0.5).

To obtain a better comprehension of the impact of g-C_3_N_4_ on the functional trait of TRGs during the removal of TC in sediment, the function level metabolism of eggNOG (evolutionary genealogy of genes: Non-supervised Orthologous Groups) category of the four sediment samples were performed and were depicted in Figure 8A and Supplementary Table 10. Obviously, the number of total predicted functional genes was higher in pig farm sediment than that in riverbed sediment (about 4.7 times higher). Overall, the predominant functional assignment across the whole dataset were mainly related to “Translation, ribosomal structure and biogenesis,” “Defense mechanism,” “Amino acid transport and metabolism,” “Inorganic ion transport and metabolism,” and “Carbohydrate transport and metabolism.” In addition, the abundance of the TRGs related to “Defense mechanism” were 30.37 and 29.89% in YCK and YT_*H*_P_*H*_, respectively, which were higher than those in HCK (24.41%) and HT_*H*_P_*H*_ (22.93%). The genes related to “Amino acid transport and metabolism” were less prevalent in pig farm sediments than that in riverbed sediments. Relative abundance of genes related to “Carbohydrate transport and metabolism” and “Translation, ribosomal structure and biogenesis” were more prevalent in pig farm sediments than those in riverbed sediments. Additionally, for the above four functional genes, g-C_3_N_4_ treatment had very slight effect on the abundance of the other three functional genes except increasing the abundance of the functional genes related to “Translation, ribosomal structure and biogenesis.”

As shown in Figure 8B, the majority of the TRGs and bacterial metabolism function presented clearly strong correlations (correlation coefficient > 0.5), the interactions between TRGs and bacterial metabolic functions were complex, 29 nodes and 79 edges were observed from the interaction of TRGs with “Translation, ribosomal structure and biogenesis,” and 27 nodes and 81 edges were observed from the interaction between TRGs and “Defense mechanism.” Among these correlations, the correlation between *tetA(58)* and some of bacterial metabolism function had the most remarkable edges and the highest betweenness centrality. There were 5, 2, 6, and 6 edges of significant and strong positive correlations (*p* < 0.05) were identified between *tetA(58)* and “Amino acid transport and metabolism,” “Carbohydrate transport and metabolism,” “Translation, ribosomal structure and biogenesis,” and “Defense mechanisms,” respectively. Compared with other TRGs, *tetA(58)* had a higher degree centrality, closeness centrality, betweenness centrality and abundance in sediment environment, indicating it was very important in the structure of networks (Supplementary Figure 4). Overall, *tetA(58)* had high relative abundance in the sediment, and strongly related with most bacterial communities and metabolism functions. Therefore, *tetA(58)* had great contribution to the shifts in bacterial metabolism functions.

## Discussion

The main active substances in the g-C_3_N_4_ photocatalytic process might be h^+^, and •O_2_^–^ (Yang and Bian, 2020; Zheng et al., 2021). Photogenerated holes (h^+^) and superoxide radical (•O_2_^–^) are the main active species for TC oxidative degradation, but hydroxyl free radicals are not the main oxidizing substances, and the influence order of active species is h^+^ > •O_2_^–^ > •OH (Liu et al., 2022). The removal efficiency of TC residues was lower in pig farm sediment than that in riverbed sediment (Figure 3), reflecting the TC removal capacity of sediment itself and the photocatalytic degradation capacity of g-C_3_N_4_. The reasons were mainly resulted from (a): The pig farm sediment had more complex nutrients than riverbed sediment, such as proteins, sugars and polyunsaturated fatty acids, which could provide more carbon sources for microorganisms. Perrin et al. (2020) pointed out that when multiple carbon sources were available, microorganisms metabolize them sequentially and some substances would be used preferentially. As a carbon source with poor bioavailability, TC would not be easily metabolized by microorganisms, especially in pig farm sediment where there were some other carbon sources that could be metabolized preferentially. (b) The removal efficiency of TC residues by g-C_3_N_4_ could be influenced by sediment environment conditions and characteristics of transmission light. The low transmittance of pig farm sediment might be due to the decrease of g-C_3_N_4_ light absorption efficiency due to the increased turbidity of pig farm sediment (Zhong et al., 2020; Sun et al., 2022b). Consequently, less light energy was available for g-C_3_N_4_ to degrade TC in pig farm sediments (Shi et al., 2021). Moreover, g-C_3_N_4_ often possesses small specific surface area and serious charge recombination, which limits its application in sediments (Liu et al., 2022; Shi et al., 2022).

Microorganisms was the most abundant group of organisms on earth and played an important role in maintaining soil biological activity and removing contaminants from the environment (Yan et al., 2020). The diversity and richness of microbial in sediment were an important indicator of environmental pollution. The bacterial diversity and richness were usually higher in most of uncontaminated sites of riverbed sediment than in contaminated sediment (Wang et al., 2015). The microbiota had higher richness and diversity in soil irrigated by river water than in soil irrigated by wastewater (He et al., 2019). In our study, the bacterial diversity and abundance in riverbed sediment were higher than those in pig farm sediment (Supplementary Tables 3, 4). These differences might mainly be attributed to (a): Bacterial growth was strongly dependent on nutrient conditions. The complex components in the pig farm sediment were not only used as nutrients for some bacteria but also used to induce toxic effects on other bacteria for inhibiting their growth and production (Cerro-Galvez et al., 2019). (b) the amounts of nutrients were different between the two sediment types, especially for organic matter and NH_4_^+^-N, (Supplementary Table 1), which might be the main factors in affecting bacteria diversity (Huang et al., 2016; Zhai et al., 2021). (c) TC concentration might be another key factor influencing the microbial community compositions of the two types of sediment samples (Gao et al., 2020). Moreover, the presence of TC altered the microbial communities and diversity, and microbial abundance also was associated with its ability to acquire ARGs (Zhang M. Q. et al., 2019). In the environment with excess nutrients, bacterial communities were able to remain a stable status for a long time despite of the perturbations of external conditions (Kearns et al., 2016). Islam and Gilbride (2019) reported that the bacterial community of swine sediment were diverse and remained stable during the TC treatment experiment. In the current study, we found that the addition of TC, g-C_3_N_4_ or TC/g-C_3_N_4_ had more obvious effect on bacterial communities’ structure in riverbed sediment than in pig farm sediment. Since the pig farm sediment possessed much more nutrients, it could effectively resist the impact of external perturbation on bacterial diversity and, thus, the diversity changes were not obvious in this sediment type. In contrast, the nutrients were inadequate in riverbed sediments and the richness of microbial community increased in response to resist the perturbations of external conditions. The microbial community might reach to a new stability in the environment (Bai et al., 2019). Therefore, TC, g-C_3_N_4_ or TC/g-C_3_N_4_ significantly increased the richness and diversity of the bacterial community in riverbed sediment type (Tang et al., 2021).

The network stability was normally strongly correlated with the complexity of molecular ecological networks (Dubey et al., 2021; Yuan et al., 2021). If the bacterial community network was simplified, the stability of the bacterial community might be reduced (Dubey et al., 2021). Previous study revealed that the accumulation of nutrients enhanced positive interaction between bacterial communities but reduced the stability of sediment ecosystem (Zhang L. L. et al., 2021). However, our research showed that pig farm sediment had abundant nutrition substances and had a significant complex bacterial network (Figure 4B). Therefore, TC, g-C_3_N_4_ and TC/g-C_3_N_4_ application had less effects on the bacterial community structure in pig farm sediment.

Studies had found that *Firmicutes* and *Bacteroidetes* were the dominant bacteria in pig manure (Luo et al., 2020). Xie et al. (2021) studied the microbial composition of riverbed sediment at different geographical locations and found that *Proteobacteria* was the most dominant bacterial flora, orderly followed by *Actinobacteria* and *Firmicutes* (Figure 5). The finding was consistent with our results for dominant microorganisms in both sediments. The relative abundances of *Acidobacteriota* in the g-C_3_N_4_ treatment was higher than the original communities. *Acidobacteriota* might be a significant component of the natural environment and played significant ecological roles in key carbon, nitrogen, and sulfur biogeochemical circuits (Kalam et al., 2020). Our result confirmed that g-C_3_N_4_ would be beneficial to the metabolism of certain microorganisms in riverbed sediment. Recently, photocatalysis had been found to exhibit remarkable antibacterial activity against some of bacterial under light and dark conditions (Balapure et al., 2020). Our study illustrated that for top ten dominant genera, only two genera, *Anaeromyxobacter* and *norank_f__Geobacteraceae* were in low abundance and have been significantly inhibited by g-C_3_N_4_ in riverbed sediment (Figure 5Bb). This suggests that g-C_3_N_4_ might produce weak toxicity in some low-abundance species, and had no obvious adverse effects on high-abundance species. In a word, when g-C_3_N_4_ degraded TC, the bacterial community structure in the two sediment types was basically stable, indicating that g-C_3_N_4_ did not obvious adverse impact on the bacterial community.

ARGs might have higher abundance in the presence of antibiotics (Zhang et al., 2020; Liu C. et al., 2021). Our research showed that ARGs such as Multidrug, TC and MLS resistance genes were much higher in pig farm sediment than in riverbed sediment (Supplementary Figure 8). Numerous studies showed that the increase of TC concentration in the environment contributed to the generation and transmission of TRGs (Gao et al., 2018; Zhang M. Q. et al., 2019). Hence, the higher prevalence and a more diverse repertory of virulence and ARGs in TC polluted samples (Jia et al., 2018). g-C_3_N_4_ treatment not only decreased the abundance of TRGs, but also reduced the occurrence and diversity of non-TC ARGs (Figure 6). Under the illumination of visible light, g-C_3_N_4_ could be involved in the oxidation/reduction of various organic substances, which significantly decreased the TC concentration as well as the level of ARGs and TRGs. Three phyla (*Actinobacteria*, *Proteobacteria*, and *Euryarchaeota*) showed strong positive correlations with different TRGs (Figure 7A), suggesting that these phyla should be the dominant hosts for these TRGs. g-C_3_N_4_ could reduce the relative abundance of TRGs by decreasing the relative abundance of potential hosts of TRGs (Figure 7B). These results were well consistent with the findings obtained by other researchers (Wang et al., 2019; Tang et al., 2021). Luo et al. (2021b) reported that photocatalyst could effectively remove TC in sediment through photocatalysis which might weaken the transport of TC from inside microbial cells, and result in the decrement of antibiotic efflux pump genes [*tetA(58)*] in sediment. Their results were further confirmed by our study, since g-C_3_N_4_ could reduce the abundance of *tetA(58)* in sediment. Thereby, like other photocatalysts (Yu et al., 2020; Gonzalez-Gaya et al., 2021), g-C_3_N_4_ could lower the abundance of bacteria with TRGs in the environment and reduced the risk of TRGs transmission in the environment.

Our results demonstrated the pig farm sediment contained more TRGs related to “Amino acid transport and metabolism” and “Defense mechanism” than riverbed sediment. Previous studies showed that amino “Amino acid transport and metabolism” function was correlated with TRGs (Wang et al., 2019; Zhang T. T. et al., 2021). Therefore, this metabolic function was extremely vital for bacteria to counteract the detrimental influence of antibiotic like TC (Zhang T. T. et al., 2021). The complex bacterial network structure and rich nutrients in the pig farm sediment made it have a strong “Defense mechanism.” These results implied the potential effect of TRGs on the metabolic functions of the specific communities in different sediment (Shen et al., 2020). Meanwhile, g-C_3_N_4_ treatment also affected the metabolic function of the sediment bacterial community (Hou et al., 2020). It is because g-C_3_N_4_ could damage bacterial cell membranes by destroying the structures of nucleic acids, proteins and other macromolecules embedded in the membranes, thus affecting function of bacterial metabolic (Zhang M. Q. et al., 2019; Zhang T. T. et al., 2021), especially in “Translation, ribosomal structure and biogenesis” (Figure 8A).

Tetracycline efflux pump genes [*tetA(58)*], which had the largest proportion of TRGs, was found in the riverbed sediment and pig farm sediment samples (Figures 6, 7). Moreover, antibiotic efflux pump genes [*tetA(58)*] had stable and positive connectivity with metabolic function of bacteria (Luo et al., 2021b). Classifying TRGs by aligning them to the eggNOG, we found that *tetA(58)* had significant and strong correlations with bacterial metabolism functions, especially with “Translation, ribosomal structure and biogenesis” and “Defense mechanisms,” indicating that *tetA(58)* was the dominant TRGs of functional metabolism in bacterial communities. The relative abundance of *tetA(58)* in g-C_3_N_4_ treatment was lower than in the CK samples, suggesting the bacterial cell membranes destruction induced by g-C_3_N_4_ increased the permeability of bacterial cells and caused the TRGs being released and eliminated (Luo et al., 2021a). Thus, g-C_3_N_4_ affected the metabolic function of bacteria and played a positive role in the removal of TRGs.

## Conclusion

This study investigated the responses of TRGs and microbial community in two sediment types against exposure of TC, g-C_3_N_4_ and TC/g-C_3_N_4_. g-C_3_N_4_ could effectively remove TC residues in pig farm sediment and riverbed sediment, with a high removal efficiency of TC in riverbed sediment type. The microbial community was shifted under the TC, g-C_3_N_4_ and TC/g-C_3_N_4_ exposure in riverbed sediment type, especially *Acidobacteriota*, *Actinobacteriota*, and *Desulfobacterota*. On the contrary, no significant change was found in the microbial community in pig farm sediment type under the TC, g-C_3_N_4_ and TC/g-C_3_N_4_ exposure. It was because there was a complex bacterial network in the pig farm sediment environment. In general, g-C_3_N_4_ treatment had little effect on the structure of the bacterial community in the two sediments, and the community structure was basically stable, especially in the pig farm sediment. Meanwhile, g-C_3_N_4_ treatment not only decreased the abundance of TRGs, but also reduced occurrence and diversity of non-TC ARGs, due to the inhibitory effects of g-C_3_N_4_ on the growth of related resistant bacteria. Interestingly, TRGs were significantly associated with metabolic functions of bacteria. Therefore, g-C_3_N_4_ could affect bacterial related metabolic functions by affecting TRGs and contributed to the removal of TRGs. Our results suggested that g-C_3_N_4_ was an environmentally friendly photocatalyst and had no obvious adverse effect on the bacterial community and the abundance of TRGs in the sediments. This study could provide some new insights for g-C_3_N_4_ for removal of TC contaminants from sediment.

## Data availability statement

All data generated or analysed during this study are included in this published article and its supplementary information files. The datasets presented in this study can be found in online repositories. The names of the repository/repositories and accession number(s) can be found below: https://www.ncbi.nlm.nih.gov/, PRJNA760830.

## Author contributions

XH designed the sampling and experiments and drafted the manuscript. XC, YT, and ZX performed the experiment and manuscript preparation. YeZ, YWa, and YuZ performed the data analyses. YWu and GW helped perform the analysis with constructive discussions and revisions of text passages. All authors commented on an early draft of the manuscript.
