# Supplementary material for: Effects of g-C3N4 on bacterial community and tetracycline resistance genes in two typical sediments in tetracycline pollution remediation
Source: Front Microbiol. 2022 Sep 16;13:964401. doi: 10.3389/fmicb.2022.964401 (PMC9523246; doi:10.3389/fmicb.2022.964401)
Supplement: Supplementary file 1 [file Data_Sheet_1.pdf]

Effects of g-C_3N_4 on bacterial community and tetracycline resistance genes in two typical sediments in tetracycline pollution remediation

Supplementary material for *Frontiers in Microbiology*

Xuemei Hu[1], Xiaoyong Chen[2], Yao Tang[1], Zhenggang Xu[3], Yelin Zeng[1], Yonghong Wang[1], Yunlin Zhao[1], Yaohui Wu[1, *], Guangjun Wang[1, **]

1. College of Life Science and technology, Central South University of Forestry and Technology, 410004, Changsha, China;

2. College of Arts and Sciences, Governors State University, University Park, IL 60484, USA;

3. Key Laboratory of National Forestry and Grassland Administration on Management of Western Forest Bio-Disaster, College of Forestry, Northwest A and F University, No.3 Taicheng Road, Yangling, Shaanxi, 712100, China;

* Corresponding author: Yaohui Wu

E-mail: wyh752100@163.com

Tel: +8613875880519

** Corresponding author: Guangjun Wang.

E-mail: csuftwgj@163.com

Tel: +86158 7409 8296

Experiment section

1. Physical and chemical properties of sediment

The sediment samples were homogenized and their physical and chemical properties were tested (Bao., 2000), sediment pH was measured in a 1:2.5 soil/water ratio solution with a compound electrode (pH glass electrode-saturated calomel electrode). The soil total nitrogen (TN), ammonium nitrogen (NH_4^+-N) and nitrate nitrogen (NO_3^--N) were measured by an Elemental analyzer (Vario MACRO cube, Elementar, Germany). The organic matter in the two soils was titrated by oil bath - potassium dichromate. Total phosphorus (TP) was determined by sodium hydroxide fusion-Molybdenum-antimony resistance colorimetric method. Total potassium (TK) was determined by flame photometry (Flame photometer 410, sherwood).

2. 16S rRNA sequencing

2.1 PCR amplification

The PCR amplification of 16S rRNA gene was performed as follows: initial denaturation at 95 ℃ for 3 min, followed by 30 cycles of denaturing at 95 ℃ for 30 s, annealing at 55 ℃ for 30s and extension at 72 ℃ for 45 s, and single extension at 72 ℃ for 10min, and end at 4 ℃. The PCR mixtures contain 5 × *TransStart* FastPfu buffer 4 μL, 2.5 mM dNTPs 2 μL, forward primer (5 μM) 0.8 μL, reverse primer (5 μM) 0.8 μL, *TransStart* FastPfu DNA Polymerase 0.4 μL,template DNA 10 ng, and finally ddH_2O up to 20 μL. PCR reactions were performed in triplicate. The PCR product was extracted from 2% agarose gel and purified using the AxyPrep DNA Gel Extraction Kit (Axygen Biosciences, Union City, CA, USA) according to manufacturer's instructions and quantified using Quantus Fluorometer (Promega, USA).

2.2 Illumina MiSeq sequencing

Purified amplicons were pooled in equimolar and paired-end sequenced (2×300) on an Illumina MiSeq PE300 platform (Illumina, San Diego, USA) according to the standard protocols by Majorbio Bio-Pharm Technology Co. Ltd. (Shanghai, China).

2.3 Processing of sequencing data

The raw 16S rRNA gene sequencing reads were demultiplexed, quality-filtered by fastp version 0.20.0 (Chen et al., 2018) and merged by FLASH version 1.2.7 (Magoc et al., 2011) with the following criteria: (i) the 300 bp reads were truncated at any site receiving an average quality score of < 20 over a

50 bp sliding window, and the truncated reads shorter than 50 bp were discarded, reads containing ambiguous characters were also discarded; (ii) only overlapping sequences longer than 10 bp were assembled according to their overlapped sequence. The maximum mismatch ratio of overlap region is 0.2. Reads that could not be assembled were discarded; (iii) Samples were distinguished according to the barcode and primers, and the sequence direction was adjusted, exact barcode matching, 2 nucleotide mis match in primer matching.

Operational taxonomic units (OTUs) with 97% similarity cutoff were clustered using UPARSE (Edgar., 2013) (Version 7.1, http://drive5.com/uparse/), and chimeric sequences were identified and removed. The taxonomy of each OTU representative sequence was analyzed by RDP Classifier (Wang et al., 2007) against the 16S rRNA database (eg. Silva SSU138) using confidence threshold of 0.7.

3. Metagenomic sequencing

3.1 Sequence quality control and genome assembly

Using the E.Z.N.A.® Soil DNA Kit (Omega Bio-tek, Norcross, GA, USA) according to manufacturer's instructions. Concentration and purity of extracted DNA was determined with TBS-380 and NanoDrop2000, respectively. DNA extract quality was checked on 1% agarose gel.

DNA extract was fragmented to an average size of about 400 bp using Covaris M220 (Gene Company Limited, China) for paired-end library construction. Paired-end library was constructed using NEXTFLEX® Rapid DNA-Seq (Bioo Scientific, Austin, TX, USA). Adapters containing the full complement of sequencing primer hybridization sites were ligated to the blunt-end of fragments. Paired-end sequencing was performed on Illumina NovaSeq/Hiseq Xten (Illumina Inc., San Diego, CA, USA) at Majorbio Bio-Pharm Technology Co., Ltd. (Shanghai, China) using NovaSeq Reagent Kits/HiSeq X Reagent Kits according to the manufacturer's instructions.

Metagenomics data were assembled using MEGAHIT (Noguchi et al., 2006) (Version 1.1.2), which makes use of succinct de Bruijn graphs. Contigs with the length being or over 300 bp were selected as the final assembling result, and then the contigs were used for further gene prediction and annotation.

3.2 Gene prediction, taxonomy, and functional annotation

Open reading frames (ORFs) from each assembled contig were predicted using MetaGene (Fu et al., 2012). The predicted ORFs with length being or over 100 bp were retrieved and translated into amino acid sequences using the NCBI translation table. A non-redundant gene catalog was constructed using

CD-HIT with 90% sequence identity and 90% coverage (Fu et al., 2012). Reads after quality control were mapped to the non-redundant gene catalog with 95% identity using SOAPaligner (Li et al., 2008), and gene abundance in each sample were evaluated.

Antibiotic resistance annotation was conducted using Diamond (Buchfink et al., 2015) (http://www.diamondsearch.org/index.php, Version 0.8.35) against CARD database (https://card.mcmaster.ca/home) with an e-value cut off of $1e^{-5}$.

Representative sequences of non-redundant gene catalog were aligned to NCBI NR database with e-value cutoff of $1e^{-5}$ using Diamond (Buchfink et al., 2015) (http://www.diamondsearch.org/index.php, Version 0.8.35) for taxonomic annotations. Cluster of orthologous groups of proteins (COG) annotation for the representative sequences was performed using Diamond (Buchfink et al., 2015) (http://www.diamondsearch.org/index.php, Version 0.8.35) against eggNOG database with an e-value cutoff of $1e^{-5}$.

Supplementary Table

Table. S1. Main physicochemical parameters measured in riverbed sediment and pig farm sediment

Sample	pH	TN [a] ($g \cdot kg^{-1}$)	TP [b] ($g \cdot kg^{-1}$)	TK [c] ($g \cdot kg^{-1}$)	SOM [d] ($g \cdot kg^{-1}$)	NH_4^+-N [e] ($mg \cdot kg^{-1}$)	NO_3^--N [f] ($mg \cdot kg^{-1}$)	TC [g] ($ng \cdot kg^{-1}$)
H	7.72	1.04	0.86	20.0	20.3	2.69	0.02	17.46
Y	7.15	13.85	43.16	8.80	201.10	1069.6	1.44	185.3

[a] TN: Total nitrogen, [b] TP: Total phosphorus, [c] TK: Total potassium, [d] SOM: Sediment organic matter , [e] NH_4^+-N: Ammonium nitrogen, [f] NO_3^--N: Nitrate nitrogen,

[g] TC: Tetracycline

Table. S2. Analytical method validation of TC detection in sediment

Name	Sediment		Recovery (%)
	LOD ($ng \cdot kg^{-1}$)	LOQ ($ng \cdot kg^{-1}$)	
TC	2.25	7.5	95.53-105.27

[a] LOD: Limits of detection, [b] LOQ: Limit of quantification

Table. S3. The number of microbial groups at each classification level of the sediment

Sample	Phyla	Classes	Order	Families	Genera	Species	OTU	Sequence numbers
**YCK**	39	101	226	357	675	1115	1696	41414
**YT_L**	43	113	239	376	683	1129	1737	48644
**YT_M**	41	109	239	381	690	1142	1777	55381
**YT_H**	45	117	251	406	748	1260	2008	60947
**YP_L**	45	111	236	378	678	1108	1701	52116
**YP_M**	42	113	247	389	691	1155	1818	52833
**YP_H**	45	115	245	384	710	1196	1863	55924
**YT_LP_L**	42	108	242	378	697	1166	1813	62971
**YT_MP_M**	44	121	256	413	756	1268	2026	59135
**YT_HP_H**	39	111	239	382	702	1165	1824	57400
**HCK**	40	124	270	393	681	1300	3118	43544
**HT_L**	41	142	314	466	783	1460	3616	47942
**HT_M**	43	143	316	469	795	1527	3850	52070
**HT_H**	43	138	311	456	774	1500	3750	52708
**HP_L**	45	139	300	435	713	1341	3304	33433
**HP_M**	43	140	307	444	730	1431	3618	43002
**HP_H**	44	140	312	458	786	1501	3784	48938
**HT_LP_L**	42	134	296	437	730	1432	3628	42688
**HT_MP_M**	43	138	303	443	741	1432	3547	42217
**HT_HP_H**	43	132	304	452	757	1457	3726	50532

Table. S4. Alpha diversity index of bacterial community in the sample

Samples	Sob	Shannon	Simpson	Chao 1
**YCK**	1696	5.09	0.0282	2279.62
**YT_L**	1737	5.14	0.0237	2348.82
**YT_M**	1777	4.90	0.0306	2429.75
**YT_H**	2008	5.18	0.0245	2724.95
**YP_L**	1710	4.94	0.0289	2314.12
**YP_M**	1818	5.00	0.0292	2707.56
**YP_H**	1863	4.99	0.0311	2524.01
**YT_LP_L**	1813	4.94	0.0298	2424.79
**YT_MP_M**	2026	5.21	0.0233	2879.16
**YT_HP_H**	1824	4.95	0.0321	2447.71
**HCK**	3118	6.59	0.0024	4227.06
**HT_L**	3616	6.85	0.0028	4711.10
**HT_M**	3850	6.87	0.0034	4971.36
**HT_H**	3750	6.86	0.0031	5068.42
**HP_L**	3304	6.84	0.0028	4563.89
**HP_M**	3618	6.87	0.0028	4939.65
**HP_H**	3784	6.85	0.0032	4878.93
**HT_LP_L**	3628	6.82	0.0038	4853.07
**HT_MP_M**	3547	6.87	0.0026	4641.98
**HT_HP_H**	3726	6.86	0.0028	4903.25

Table. S5. Relative abundances of dominant bacterial phyla of pig farm sediment under different treatments

Sample	*Firmicutes*	*Bacteroidota*	*Proteobacteria*	*Actinobacteriota*	*Chloroflexi*	*Desulfobacterota*	*Myxococcota*	*Patescibacteria*	*Spirochaetota*	*Deinococcota*	others
**YCK**	46.82%	13.59%	11.78%	11.34%	4.04%	2.39%	1.88%	1.05%	0.91%	0.61%	5.59%
**YT_L**	36.90%	19.73%	11.14%	8.83%	5.48%	4.02%	2.26%	1.91%	0.88%	0.71%	8.14%
**YT_M**	44.32%	15.98%	12.55%	9.40%	3.46%	3.60%	2.23%	0.78%	1.08%	0.67%	5.93%
**YT_H**	37.15%	19.36%	12.68%	7.38%	5.74%	4.19%	2.18%	1.18%	1.59%	0.92%	7.63%
**YP_L**	39.08%	22.63%	13.54%	7.42%	3.01%	4.57%	2.26%	1.15%	0.63%	1.04%	4.67%
**YP_M**	39.64%	18.42%	12.69%	8.27%	4.97%	4.97%	2.09%	1.24%	1.00%	0.99%	5.71%
**YP_H**	38.87%	20.44%	10.71%	7.73%	4.72%	4.79%	2.03%	1.45%	0.69%	1.15%	7.42%
**YT_LP_L**	44.13%	16.86%	9.38%	8.84%	4.47%	5.57%	1.93%	1.10%	2.11%	0.64%	4.97%
**YT_MP_M**	36.77%	19.86%	11.33%	8.05%	4.96%	4.48%	2.31%	1.61%	2.03%	1.23%	7.37%
**YT_HP_H**	43.23%	16.98%	11.77%	8.67%	5.67%	2.96%	2.67%	0.67%	0.81%	1.17%	5.4%

Table. S6. Relative abundances of dominant bacterial phyla of riverbed sediment under different treatments

Sample	*Acidobacteriota*	*Proteobacteria*	*Actinobacteriota*	*Chloroflexi*	*Firmicutes*	*Gemmatimonadota*	*Myxococcota*	*Cyanobacteria*	*Desulfobacterota*	*Methylomirabilota*	Others
**HCK**	16.07%	17.77%	25.05%	11.64%	8.68%	3.27%	3.42%	3.50%	1.70%	1.79%	7.11%
HT_L	12.78%	20.70%	20.37%	9.82%	6.37%	5.75%	4.98%	2.12%	4.42%	2.36%	10.33%
HT_M	14.53%	17.55%	18.83%	12.50%	6.84%	4.02%	4.67%	4.98%	3.46%	2.08%	10.53%
HT_H	15.10%	17.55%	20.37%	13.94%	4.49%	4.94%	4.09%	2.59%	3.34%	2.76%	10.83%
HP_L	24.03%	18.66%	17.47%	11.01%	4.63%	5.39%	3.50%	0.88%	1.51%	2.22%	10.70%
HP_M	20.41%	20.89%	16.59%	10.70%	3.64%	4.82%	3.80%	3.00%	1.10%	2.78%	12.27%
HP_H	18.24%	19.85%	17.49%	12.50%	5.00%	3.39%	3.17%	6.34%	1.54%	1.81%	10.67%
HT_LP_L	22.53%	17.45%	16.28%	11.99%	5.61%	3.23%	3.21%	4.96%	1.29%	1.74%	10.71%
HT_MP_M	20.44%	19.54%	16.42%	11.94%	4.85%	5.32%	3.66%	2.76%	1.78%	1.84%	11.45%
HT_HP_H	22.03%	18.06%	17.46%	12.70%	4.47%	5.03%	3.60%	2.16%	1.42%	2.12%	10.95%

Table. S7. Sequence assemblies and gene carrying contigs for metagenomic sequencing

Samples	Clean reads	Clean base (bp)	Percent in raw reads (%)	Percent in raw bases (%)	contigs	N50 (bp)	N90 (bp)	ORFs
**YCK**	64001340	9634880228	98.75550304	98.45586942	740704	770	358	11002877
YT_HP_H	55466256	8355956980	98.66726342	98.43815819	634697	837	361	867203
**HCK**	64790898	9771089218	97.83780393	97.71443566	55822	509	334	672221
HT_HP_H	57283390	8636814739	97.90654423	97.75965653	402528	502	332	481248

Table. S8. The frequency of ARGs in sediment samples by antibiotic class

Antibiotic class	YCK	YT_HP_H	HCK	HT_HP_H
Multidrug	371808	323246	105128	84020
Tetracycline	134216	103702	27090	22388
MLS	112524	93904	28106	21090
Glycopeptide	78478	81116	21458	18216
Peptide	61492	49434	14124	11210
Aminocoumarin	47478	32866	13200	10614
Mupirocin	30824	24856	5138	4464
Beta-lactam	22614	24742	5466	4528
Fluoroquinolone	27232	16634	6330	4604
Pleuromutilin	20092	15570	4244	3324
Aminoglycoside	17642	15232	3588	2840
Sulfonamide	17554	11778	1368	974
Rifamycin	13622	7282	3306	3038
Phenicol	13914	10772	706	706
Fosfomycin	8972	4958	2164	1688
Triclosan	4948	3838	1214	858
Diaminopyrimidine	3054	2276	330	318
Bicyclomycin	2904	1518	360	408
Elfamycin	1528	608	922	878
Nucleoside	276	152	2	0
Total	991172	824484	244244	196166

Table. S9. The abundances of 50 background ARGs in 4 sediment samples

Antibiotic class	ARO name	YCK	YT_HP_H	HCK	HT_HP_H
Tetracycline	*tetA(58)*	49106	32054	11316	9054
	otr(A)	9280	11710	2582	2046
	tetB(P)	10224	8882	1710	1390
	tetT	6756	6078	1124	1134
	tetA(46)	6212	4478	1236	1132
Aminocoumarin	*novA*	27798	18670	7166	5882
	parY	19680	14196	6034	4732
Aminoglycoside	*kdpE*	4438	6056	2520	1876
Fluoroquinolone	*patB*	8946	6724	1798	1488
	patA	7898	4692	2080	1430
Glycopeptide	*vanRF*	5536	7818	2222	1788
	vanRM	4594	4380	1880	1600
	vanHF	5280	5042	512	420
	vanSA	5224	4050	674	590
MLS	*macB*	61782	51240	17878	13566
	oleC	18570	13458	6422	4606
	lmrD	7110	6784	1304	856
Multidrug	*evgS*	33042	42904	6160	5106
	msbA	20632	15410	4852	3932
	rpoB2	14900	12688	6384	5902
	mtrA	13792	10086	5586	4604
	baeS	12144	9306	3508	2940
	smeS	8734	9128	4702	3646
	lmrC	12356	8940	1940	1724
	arlS	8816	11482	2674	1882
	efrA	11790	7928	2538	2088
	optrA	10306	8486	2368	2040
	arlR	9338	9426	2488	1674
	cpxA	7692	7434	2174	1868
	MexW	6252	5700	2202	1916
	carA	6410	4600	2678	2264
	mdtB	5572	7406	1058	888
	tlrC	5772	4336	2294	1644
	efrB	6426	3914	1762	1408
	mdtC	4534	6004	1500	1182
	oleB	6520	3322	1756	1532
	rpoB	5298	3328	2024	1920
	srmB	5120	4040	1674	1378
	evgA	4412	3754	1844	1682
	Corynebacterium striatum tetA	4536	3516	1892	1452

Continued table

	MuxB	3730	3908	2002	1564
Mupirocin	*mupA*	18884	15960	2136	1902
	mupB	8218	6806	2462	2206
Peptide	*bcrA*	26748	21908	6768	5214
	basS	5816	4642	1640	1270
	rosB	5732	5344	616	544
Pleuromutilin	*TaeA*	20092	15570	4244	3324
Rifamycin	*rphB*	5796	3880	844	844
Sulfonamide	*sul4*	5392	4570	1198	744
	sul1	7046	3762	66	52

Table. S10. Absolute abundance of bacterial metabolic function encoded by TRGs

Function	YCK	YT_HP_H	HCK	HT_HP_H
Energy production and conversion	1607	869	703	518
Cell cycle control, cell division, chromosome partitioning	443	240	37	16
Amino acid transport and metabolism	21734	17659	2802	1725
Carbohydrate transport and metabolism	7817	5907	2600	2170
Coenzyme transport and metabolism	62	434	340	215
Translation, ribosomal structure and biogenesis	26072	23033	6925	6884
Transcription	0	0	9	7
Replication, recombination and repair	38	0	56	2
Cell wall/membrane/envelope biogenesis	6182	4591	1142	1129
Post-translational modification, protein turnover, chaperones	0	0	16	18
Inorganic ion transport and metabolism	16203	12136	3740	2723
Secondary metabolites biosynthesis, transport and catabolism	679	675	264	178
Function unknown	8247	5715	1474	1196
Signal transduction mechanisms	6861	4790	1156	1189
Intracellular trafficking, secretion, and vesicular transport	1	3	0	0
Defense mechanisms	42095	31990	6869	5349
Total	138041	108042	28133	23319

Supplementary Figure

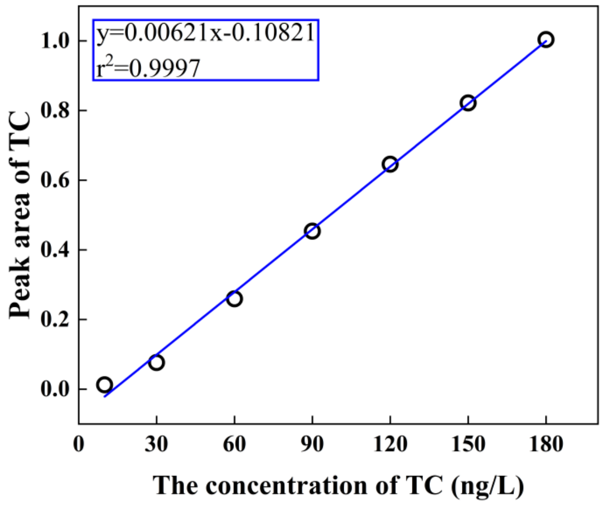

Fig. S1. Standard curve for quantification of detected TC

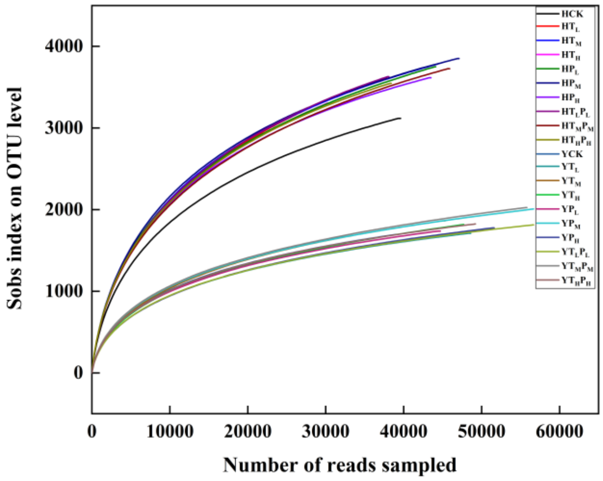

Fig. S2. Rarefaction curves for the Sob richness.

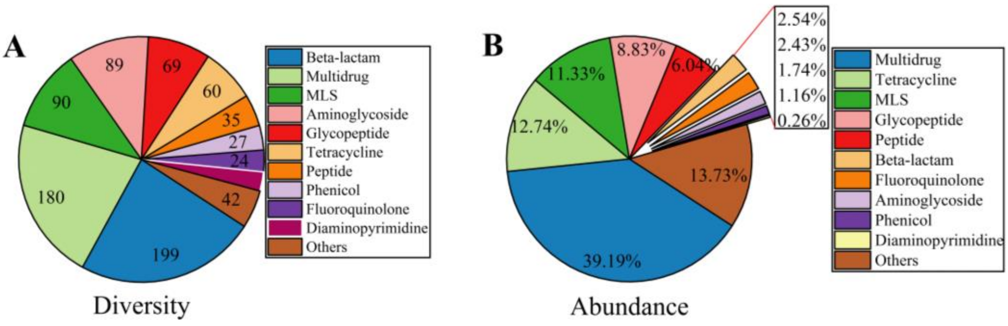

Fig. S3. Diversity and abundance of ARGs types. (A) Diversity of ARGs types, (B) Abundance of ARGs types.

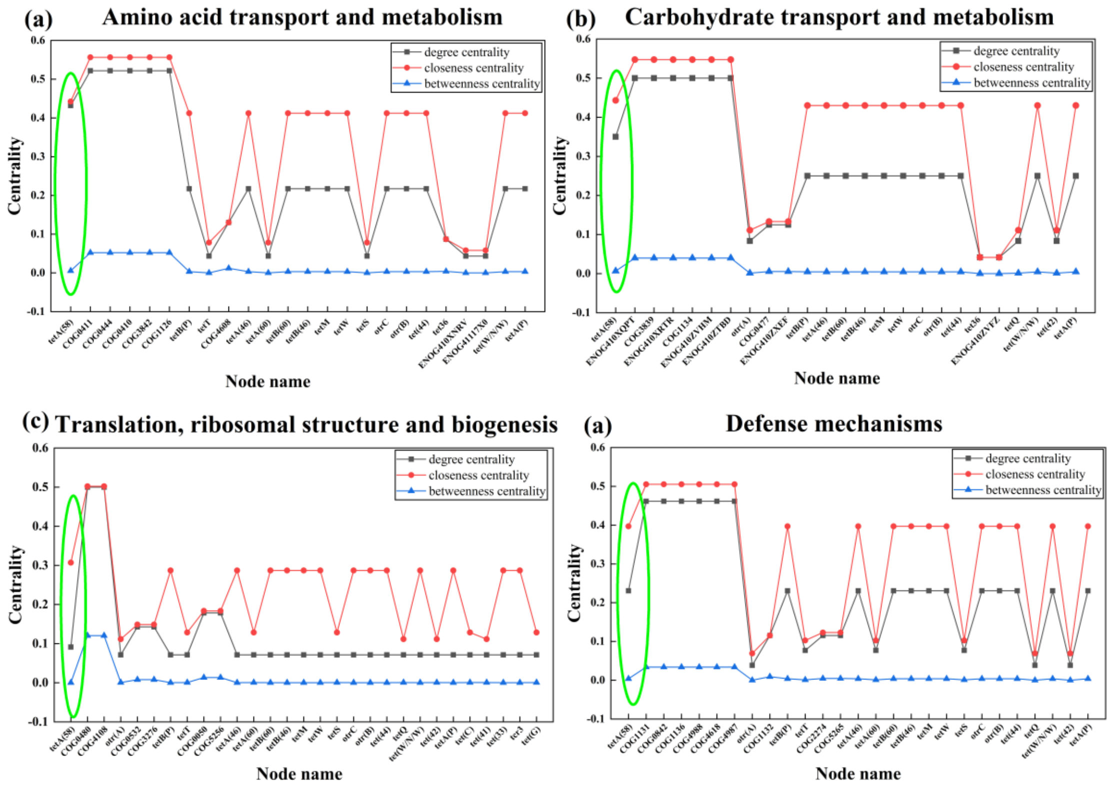

Fig. S4. Node centrality analysis on level. (a) Node centrality analysis for Amino acid transport and metabolism, (b) Node centrality analysis for Carbohydrate transport and metabolism, (c) Node centrality analysis for Translation, ribosomal structure and biogenesis, (d) Node centrality analysis for Defense mechanisms.

References

Bao, S.D., 2000. The Soil Agricultural Chemistry Analysis. *Chinese Agriculture Press* (in chinese).

Buchfink, B., Xie, C., Huson, D.H., 2015. Fast and sensitive protein alignment using DIAMOND. *Nat Methods*. 12 (1): 59-60.

Chen, S., Zhou, Y., Chen, Y., Gu, J., 2018. fastp: an ultra-fast all-in-one FASTQ preprocessor. *Bioinformatics*. 34 (17): i884-i890.

Edgar, R.C., 2013. UPARSE: highly accurate OTU sequences from microbial amplicon reads. *Nat Methods*. 10(10): 996-998.

Fu, L., Niu, B., Zhu, Z., Wu, S., Li, W., 2012. CD-HIT: accelerated for clustering the next-generation sequencing data. *Bioinformatics*. 28 (23): 3150-3152.

Li, R., Li, Y., Kristiansen, K., Wang, J., 2008. SOAP: short oligonucleotide alignment program. *Bioinformatics*. 24 (5): 713-714.

Magoč, T., Salzberg, S.L., 2011. FLASH: fast length adjustment of short reads to improve genome assemblies. *Bioinformatics*. 27 (21):2957-2963.

Noguchi, H., Park, J., Takagi, T., 2006. MetaGene: prokaryotic gene finding from environmental genome shotgun sequences. *Nucleic Acids Res*. 34 (19):5623-5630.

Wang, Q., Garrity, G.M., Tiedje, JM., Cole, J.R., 2007. Naive Bayesian classifier for rapid assignment of rRNA sequences into the new bacterial taxonomy. *Appl Environ Microbiol*. 73 (16):5261-5267.

Zhang, H., Du, M., Jiang, H., Zhang, D., Lin, L., Ye, H., 2015. Occurrence, seasonal variation and removal efficiency of antibiotics and their metabolites in wastewater treatment plants, Jiulongjiang River Basin, South China. Environ *Sci Proc Imp*. 17:225-234.
